# Nintedanib plus letrozole in early breast cancer: a phase 0/I pharmacodynamic, pharmacokinetic, and safety clinical trial of combined FGFR1 and aromatase inhibition

**DOI:** 10.1186/s13058-019-1152-x

**Published:** 2019-05-24

**Authors:** Miguel Quintela-Fandino, Juan V. Apala, Diego Malon, Silvana Mouron, Javier Hornedo, Lucia Gonzalez-Cortijo, Ramon Colomer, Juan Guerra

**Affiliations:** 1 Breast Cancer Clinical Research Unit, CNIO–Spanish National Cancer Research Center, Melchor Fernandez Almagro, 3, 28029 Madrid, Spain; 2 Medical Oncology, Hospital Universitario de Fuenlabrada, Fuenlabrada, Spain; 3 Medical Oncology, Hospital Universitario Quiron, Pozuelo de Alarcon, Spain; 4 Medical Oncology, Hospital Universitario La Princesa, Madrid, Spain

**Keywords:** Phase 0 clinical trial, Phase I clinical trial, Pharmacodynamics, FGFR1, FGF23, Letrozole, Nintedanib

## Abstract

**Background:**

The combined use of a FGFR1 blocker and aromatase inhibitors is appealing for treating breast cancer patients with FGFR1 amplification. However, no pharmacodynamic studies have addressed the effects of this combined target modulation. We conducted a phase 0/I clinical trial in an adjuvant setting, with the goal of obtaining pharmacodynamic proof of the effects of combined aromatase and FGFR1 inhibition and to establish the RP2D for nintedanib combined with letrozole.

**Patients and methods:**

Women with early-stage luminal breast cancer were eligible for enrollment in the study. Dose level 1 was nintedanib (150 mg/bid) plus letrozole (2.5 mg/day) administered for a single 28-day cycle (DLT assessment period), followed by a classic 3 + 3 schedule. FGF23 and 17-B-estradiol levels were determined on days 0 and 15; pharmacokinetic parameters were assessed on days 1 and 28. Patients were allowed to continue treatment for 6 cycles. The primary study endpoint was a demonstration of FGFR1 modulation (defined as a 25% increase in the plasma FGF23 level).

**Results:**

A total of 19 patients were enrolled in the study (10 in the expansion cohort following dose escalation). At the RP2D (nintedanib 200 mg/bid plus letrozole 2.5 mg/day), we observed a 55% mean increase in the plasma FGF23 level, and 81.2% of the patients had no detectable level of 17-B-estradiol in their plasma (87.5% of the patients treated with letrozole alone). Nintedanib and letrozole displayed a pharmacokinetic interaction that led to three- and twofold increases in their respective plasma concentrations. Most G3 toxic events (5 out of 6: 2 diarrhea and 3 hypertransaminasemia) occurred subsequent to the DLT assessment period.

**Conclusion:**

Combined treatment with nintedanib (200 mg/bid) plus letrozole (2.5 mg/day) effectively suppressed FGFR1 and aromatase activity, and these respective doses can be used as starting doses in any subsequent trials. However, drug-drug interactions may produce tolerability issues when these drugs are co-administered for an extended time period (e.g., 6 months). Patients enrolled in future trials with these drugs should be carefully monitored for their FGF23 levels and signs of toxicity, and those findings should guide individualized treatment decisions.

**Trial registration:**

This trial was registered at www.clinicaltrials.gov under reg. # NCT02619162, on December 2, 2015.

## Background

Several biological factors are known to modulate the sensitivity of hormone receptor-positive advanced breast cancer to hormonal therapy [1], which is the cornerstone for clinical management of this disease. One of these factors is the amplification of fibroblast growth factor receptor 1 (FGFR1) [2]. Retrospective studies suggest that ~ 10% of breast cancer patients harbor this amplification [2, 3]. Although FGFR1 amplification can occur in triple-negative breast cancer, it most commonly occurs in hormone receptor-positive breast cancer [2–4]. FGFR1 amplification has been linked to ligand-dependent and ligand-independent increases in MAPK and Pi3K activation and signaling [2, 5], tumor progression [6], resistance to hormonal inhibitors [6, 7], early distant disease relapse, and poor patient survival rates, despite standard-of-care treatment of loco-regional disease [3, 4].

Several small molecules that target FGFR receptors have been developed and can be divided into three main classes [6]: (1) non-selective and selective tyrosine-kinase inhibitors (TKIs), (2) monoclonal antibodies, (3) FGF ligand traps. Among these, TKIs are at the most advanced stages of development, and several of those agents have shown promising clinical efficacy when used to treat tumors that harbor activating mutations in at least one of the four FGF receptors [5, 8, 9]. Nintedanib is a TKI with activity against the FGFR, VEGFR, and PDGFR families, as well as other targets [10]. Preclinical pharmacology studies in tumor models have suggested an antiangiogenic profile for nintedanib [10–12] and that drug has shown clinical activity in cases of mesothelioma [13], lung cancer [14], and breast cancer [15]. Nintedanib is currently approved for use in combination with docetaxel for treating advanced lung cancer that has progressed after first-line chemotherapy. Preclinical studies have shown that nintedanib exerts an anti-fibrogenic effect by blocking FGFR1 in fibroblasts [16]. This effect accounts for its clinical activity in treating cases of idiopathic pulmonary fibrosis, which is one indication for which nintedanib was approved on the basis of positive results in randomized clinical trials [17, 18]. As a multi-targeted inhibitor, nintedanib has been tested for its anti-cancer effects mostly as an antiangiogenic agent; however, its activity as an FGFR1 inhibitor in oncology remains unexplored.

Non-specific and specific FGFR inhibitors have shown only moderate levels of toxicity; however, their various toxic effects become more evident with long-term use [6]. Specific FGFR inhibitors cause hyperphosphatemia, mucositis, hair modification, eye toxicity, osteoarticular toxicity, and onycholysis, whereas non-specific agents also produce other side effects such as fatigue, anorexia, gastrointestinal and liver toxicity, and VEGFR inhibition-related toxicity [6]. Interestingly, nintedanib has a favorable toxicity profile when administered alone [19] or in combination with other agents [20–22].

Selective pan-FGFR inhibitors have been used after the successful completion of dose-escalation trials in “basket” clinical trials in a histology-agnostic manner. While two large trials were recently completed, neither trial showed encouraging activity: in arm W of the MATCH trial, the response rate to AZD4547 was low (8%) and the agent’s activity was greater in tumors with FGFR gene fusions than in those with FGFR amplifications. However, the patients with FGFR amplifications showed better clinical effects (50% disease stabilization rate) than the patients with FGFR1 SNVs [23]. Another clinical trial that used Debio1347 in multiple tumor types yielded similar efficacy results [24]. A significant number of patients in both trials (32% and 21%) were breast cancer patients with FGFR amplifications. One trial that examined the efficacy of a FGFR1 inhibitor combined with the multikinase inhibitor dovitinib showed impressive clinical activity in patients who had advanced hormone receptor-positive breast cancer with FGFR1 amplification [25]. However, strong preclinical data suggests that the maximum efficacy of that intervention should be observed in when it is administered in combination with hormonal agents [2, 7]. Thus, developing the combination of nintedanib plus a hormonal agent as a treatment for FGFR1-amplified hormone receptor-positive breast cancer would be appealing, once a pharmacodynamic modulation of FGFR1 at a standard clinical dose has been demonstrated. Possible clinical settings for testing such combinations (always in FGFR1-amplified patients) include an advanced disease setting and in patients with high-risk early breast cancer in an adjuvant setting. Prior to considering a large-scale trial in either setting, we sought to better understand the potential clinical effects that might be produced by a combination of nintedanib plus letrozole in patients with hormone receptor-positive breast cancer. Specifically, we aimed to (1) determine whether nintedanib administered in combination with letrozole at the recommended phase-II dose levels (RP2D) would prove the pharmacodynamic effect of FGFR1 inhibition (i.e., an increase in plasma FGF23 levels) [26], while maintaining letrozole-induced 17-B-estradiol suppression; (2) study potential pharmacokinetic interactions between the two drugs; and (3) assess the short-term and long-term toxicity of the combination. To that end, we conducted a phase 0/I clinical trial in hormone receptor-positive breast cancer patients that had previously completed loco-regional treatment and were candidates for receiving aromatase inhibitors in an adjuvant setting. Because the patients received the experimental treatment during their adjuvant stage, no efficacy endpoints were included in this trial.

## Patients and methods

This was a prospective, open-label, multicenter, single-arm phase 0/1 investigator-initiated study. The study was conducted in accordance with the Declaration of Helsinki and Good Clinical Practice standards. Institutional Review Board approval was obtained from all the participating hospitals, and all patients provided their written informed consent prior to enrollment. The study was registered at Clinicaltrials.gov (NCT02619162).

### Study population

Females ≥ 18 years old were eligible for enrollment if they had a hormone receptor-positive breast tumor (defined as positivity for the ER and/or PR in > 5% of tumor cells tested at the local pathology department) > 1 cm in size that had been adequately treated within less than 6 months prior to registration and were amenable to receiving the aromatase inhibitor letrozole as a part of their adjuvant treatment. Patients had to be post-menopausal according to any of the following criteria: (1) women of any age who had a bilateral oophorectomy (including radiation castration and confirmed by subsequent amenorrhea for > 3 months) and were amenorrheic for > 3 months, (2) women that had spontaneous cessation of menses for ≥ 12 consecutive months, (3) women that had FSH, LH, and 17-B-estradiol levels in postmenopausal ranges without an alternative cause. A minimum of 4 weeks and a maximum of 24 weeks between the last treatment procedure (chemotherapy and/or radiation therapy; 6 weeks from major surgery) and registration were allowed. Patients had to have been taking letrozole for at least 4 weeks prior to registration. Other inclusion criteria included the following: an ECOG performance status of 0–1; adequate liver, renal, and hematologic function (defined as serum bilirubin < 1.25-fold the upper limit of normal [ULN], AST/ALT < 1.25-fold the ULN, serum creatinine < 1.5-fold the ULN or creatinine clearance < 50 mL/min, hemoglobin > 10 g/dL, a platelet count > 100 × 10^9^/L, and a granulocyte count > 1.5 × 10^9^/L), and LVEF > 50%. Patients who had not recovered from previous toxicity to a tolerable grade of ≤ 2, who had bilateral tumors, who had concurrent severe conditions, who were taking anticoagulants, or with a history of clinically significant bleeding or thromboembolic events within 6 months of study entry were excluded from the study. A CT scan showing the lack of metastatic spread was required within 4 weeks prior to trial registration (plus bone scintigraphy and/or brain MRI, if indicated).

### Study design and procedures

Patients started by taking letrozole at a fixed oral dose of 2.5 mg/day plus nintedanib bid on a continuous schedule. One treatment cycle was defined as 28 consecutive days of continuous treatment with both agents. Three dose levels of nintedanib were available for use: 150 mg/bid (level 1), 200 mg/bid (level 2), and 250 mg/bid (level 3). Dose escalation followed a classic 3 + 3 schedule. The first cycle was the dose-limiting toxicity (DLT) assessment period and was mandatory for all patients. DLT was defined as the occurrence of drug-related adverse events during the first treatment cycle; these events included grade 4 anemia or thrombocytopenia at any time; grade 4 neutropenia persisting for ≥ 5 days; grade 3 nausea, diarrhea, or vomiting in spite of maximal supportive care and prophylaxis; clinically significant grade 3 or greater non-hematologic toxicity (not including alopecia, anorexia, or fatigue); and a serum creatinine value ≥ 2-fold the baseline value or ≥ 2-fold the ULN if the baseline value was less than the ULN. The recommended phase 2 dose (RP2D) was the level at which fewer than two of a minimum of six patients experienced DLT. An expansion cohort started at the RP2D after the dose-escalation part of the study was completed.

Patients being treated at level 1 were allowed one nintedanib dose reduction (to 100 mg/bid), whereas patients at levels 2 and 3 were allowed two 50 mg/bid dose reductions. Patients requiring further nintedanib dose reductions or patients requiring letrozole dose reductions were removed for the trial. Patients were followed up on a weekly basis during cycle 1, on a bi-weekly basis during cycle two, and every 4 weeks afterwards.

**Fig. 1:**
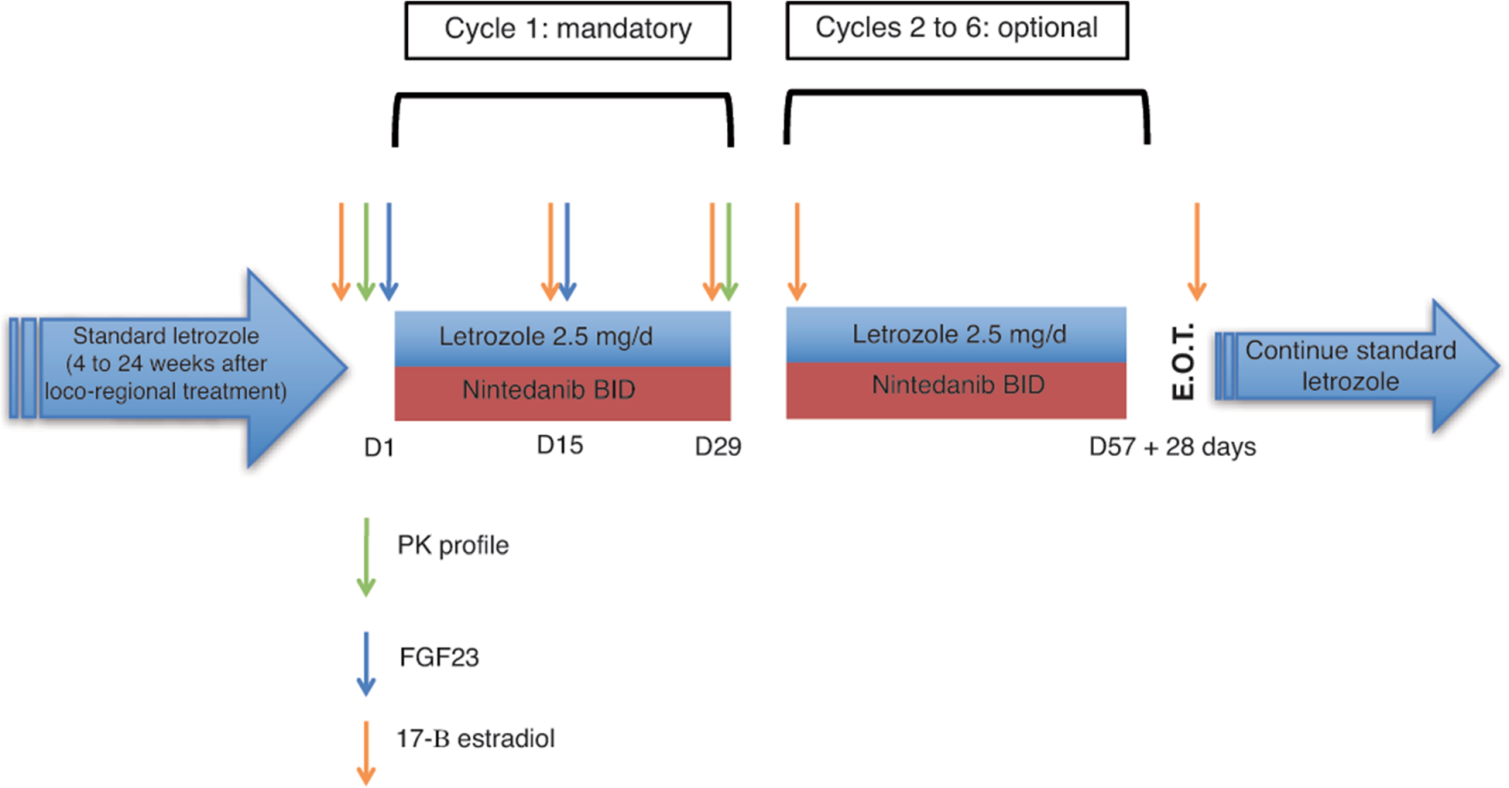
Trial design. Patients completing the treatment for early hormone receptor-positive breast cancer that were eligible for letrozole treatment and were receiving it for a minimum of 4 weeks were enrolled in the trial. This design allowed testing whether letrozole adjuvant treatment was actually achieving its therapeutic goal (17-B-estradiol suppression) and whether the concurrent administration of nintedanib exerted any negative influence on it even in the absence of pharmacokinetic interactions between both drugs. Cycle 1 was mandatory and included pharmacokinetic profiling and pharmacodynamic endpoints (FGF23 and 17-B-estradiol suppression). Cycles 2 to 6 were included in order to provide the option for any potential benefit that a long-term administration of a multi-tyrosine-kinase inhibitor could imply in the adjuvant setting, as long as patients willingly decided to continue treatment and no toxicity was observed. After completing 1 to 6 cycles, patients continued on standard adjuvant letrozole treatment. And end-of-treatment (EOT) visit was scheduled 28 days after the last nintedanib dose

Patients who had completed their first cycle of treatment in either the dose escalation or expansion cohort portion of the study without experiencing significant toxicity were allowed to continue their treatment for 5 additional cycles. Patients who completed 6 cycles (and patients coming off a trial at earlier time points because of a personal or investigator decision or significant toxicity) stopped receiving nintedanib and were given standard letrozole treatment according to current adjuvant disease management guidelines. Figure 1 depicts the basic trial design.

The primary study endpoint was the change in FGF23 levels (pg/mL) from baseline to day + 15. Secondary endpoints were (1) change in 17-B-estradiol levels (pg/mL) from baseline to day + 15, (2) toxicity graded according to NCI CTCAE V.4.03, and (3) letrozole and nintedanib pharmacokinetic parameters and interactions.

### Pharmacokinetics and pharmacodynamics

Blood samples (two 5-mL EDTA tubes per time point—one for each agent’s pharmacokinetic determinations) were collected on days 1 and 2 and again on days 29 and 30, at 0 min, 15 min, 30 min, 1 h, 2 h, 4 h, 7 h, and 24 h after taking both oral agents, and used to determine the pharmacokinetic parameters of nintedanib and letrozole. For the extraction of letrozole from human plasma, 10 μL of plasma was added to 200 μL of acetonitrile, and the mixture was centrifuged. Next, a 50-μL aliquot of the supernatant was injected into a Thermo UPLC-MS/MS system. Two species of nintedanib were identified: BIBF1120-BS (nintedanib) and its active metabolite, BIBF1202-ZW. The assay comprised a solid phase extraction step in a 96-well plate format followed by quantification by LC-MS/MS using deuterated internal standards. The samples were analyzed on a Sciex API 5000 LC-MS/MS system. An electrospray ion source (atmospheric pressure ionization (API)) was used for ionization. Measurements were taken in the positive ionization mode. Separation was accomplished by injection onto a Phenomenex Luna (C18) 100 Å, 30 × 2.0 mm (3.0 μm) HPLC column. All of the analytes were well separated by the column, and the internal standards co-eluted with their respective analytes. Thermo XCalibur and LCQuan software were used to calculate drug concentrations, and the results were used to perform pharmacokinetic analyses with the PK Solutions software package (Summit Research Services). The lower limits of detection for the pharmacokinetic assays were 1 ng/mL for letrozole, 0.05 ng/mL for BIBF1120-BS, and 0.1 ng/mL for BIBF1202-ZW.

One additional blood sample (7-mL BD Vacutainer CPT Tube) was obtained on day + 1 and again on day + 15 prior to the morning nintedanib dose and used to determine FGF23 levels. Native FGF23 was determined at the central laboratory by using a FGF23-ELISA Kit from Millipore according to the manufacturer’s instructions. Briefly, FGF23 molecules were captured from the samples in the wells of a microtiter plate that had been coated with a polyclonal goat anti-human FGF-23 antibody. A second biotinylated polyclonal goat anti-human antibody was added to the captured molecules, followed by the binding of streptavidin-horseradish peroxidase conjugate to the immobilized biotinylated antibodies. The levels of immobilized antibody-enzyme conjugates were quantified by measuring horseradish peroxidase activity in the presence of the substrate 3,3′,5,5′-tetramethylbenzidine. Enzyme activity was measured spectrophotometrically as the increase in absorbance at 450–590 nm after acidification of the formed products. All samples were assayed in triplicate, and values were expressed in picograms per milliliter.

17-B-estradiol levels were determined at each of the following time points: within 7 days after the first dose of nintedanib (“baseline”), on day 15 of cycle 1, on day 1 of every subsequent cycle, and finally at the end-of-treatment visit (EOT, 28 days after the last study dose). Those levels were determined at each study site with a clinical assay that used blood samples drawn for the trial procedures. The assay’s lower limit of detection (LLD) was 5 pg/mL.

### Statistical analysis

The sample size needed for the dose-escalation portion of the study was determined by the requirements for a classic 3 + 3 design. The sample size for the expansion cohort portion was determined by estimating the expected change in FGF23 levels after 14 days of treatment with nintedanib. When using a power of 80% and an alpha error of 5%, a minimum of 15 subjects were necessary to observe at least a 25% increase in the median plasma FGF23 level. Assuming that the nintedanib dose level would define the RP2D, we needed to recruit a minimum of six patients, and after including those patients in the pharmacodynamic analysis, an expansion cohort of ten patients was sufficient to demonstrate the primary endpoint.

All patients who received at least one dose of nintedanib plus letrozole were included in the toxicity and correlation analysis. The correlation between BIBF1120-BS AUC and FGF23 blood levels was examined using Pearson’s *R*^2^ coefficient. FGF23 and 17-B-estradiol levels were compared by using non-parametric (Wilcoxon rank) tests that were performed with SPSS V.19 software.

## Results

### Patient characteristics

From September 2015 to November 2017, 20 patients were enrolled in the study. There was 1 screening failure (baseline elevation of transaminases beyond the inclusion criteria limit), for a total number of 19 evaluable patients. Characteristics of the enrolled patients are shown in Table 1. All 19 patients were included in the toxicity, pharmacokinetic, and pharmacodynamic evaluations.

**Table 1:** Demographic and baseline clinical characteristics

Characteristic	Level	*N* (%)
Age (years; median, range)	Level 1	50.6 (50.1–51.5)
	Level 2	59.5 (53.4–69.2)
	Total	57.5 (50.1–69.2)
ECOG	Level 1	0: 3/3 (100%)
	Level 2	0: 16/16 (100%)
	Total	0: 19/19 (100%)
Tumor size	Level 1	T1: 3/3 (100%)
	Level 2	T1: 8/16 (50%); T2: 8/16 (50%)
	Total	T1: 11/19 (58%); T2: 8/19 (42%)
Nodal status	Level 1	N0: 2/3 (66.6%); N1: 1/3 (33.3%)
	Level 2	N0: 9/16 (56.2%); N1: 6/16 (37.5%); N2: 1/16 (6.2%)
	Total	N0: 11/19 (57.9%), N1: 7/19 (36.8%); N2: 1/19 (5.3%)
ER and/or PR > 5%	Level 1	3/3 (100%)
	Level 2	16/16 (100%)
	Total	19/19 (100%)
HER2	Level 1	0/3 (0%)
	Level 2	0/16 (0%)
	Total	0/19 (0%)
Grade	Level 1	G1: 3/3 (100%)
	Level 2	G1: 9/16 (56%); G2: 6/16 (38%); G3: 1/16 (6%)
	Total	G1: 12/19 (64%); G2: 6/19 (31%); G3: 1/19 (5%)
Ki67 (average, range)	Level 1	9% (3–15%)
	Level 2	11.5% (2–43%)
	Total	11.1% (2–43%)
Adjuvant/neoadjuvant chemotherapy prior to study registration	Level 1	0/3 (0%)
	Level 2	Anthracyclines plus taxanes: 5/16 (31.3%); taxanes only: 1/16 (6.2%); none: 10/16 (62.5%)
	Total	Anthracyclines plus taxanes: 5/19 (26.4%); taxanes only: 1/19 (5.3%); none: 13/19 (68.3%)
Letrozole time (days) at study registration (average, range)*	Level 1	134 (131–140)
	Level 2	117 (29–220)*
	Total	119 (29–220)*

*The trial allowed patients that had completed their adjuvant treatment and had already started letrozole for a minimum of 4 weeks. Two patients started letrozole before scheduled adjuvant radiation therapy, and because of this reason, they had been receiving letrozole for 209 and 220 days. However, since they did not receive other modality of non-standard adjuvant treatment in between, they were allowed to enter the trial despite a maximum pre-established boundary of 180 days in letrozole prior to study registration

### RP2D determination and treatment duration

Three patients were enrolled in the first level of the study, and no grade 3 or non-tolerable grade 2 toxicities were observed.

Accrual then proceeded to the second level of the study. The third patient recruited in level 2 experienced grade 3 hypertension after 3 weeks of treatment. That patient was not previously hypertensive and had no history of taking blood pressure-lowering medications; therefore, the patient’s hypertension qualified as a DLT. Three more patients were enrolled in level 2. No further DLTs were observed among those patients during the first cycle, but all 3 patients experienced at least 1 grade 1 or 2 event. At that point, after taking into account the observed DLT and existing toxicity data (particularly the high incidence of diarrhea) obtained from other studies that has used long-term administration of nintedanib at 250 mg/bid on a continuous schedule [21, 27–29], the steering committee decided to increase the number of subjects at that level. Ten more patients were accrued, and no additional patient experienced a DLT during the DLT assessment period. However, 5 out of 16 patients required a dose-reduction at that level during subsequent cycles because of grade 3 toxicity events that would have been defined as DLTs if they had occurred during the first cycle. With the exception of one grade 3 liver enzyme elevation event and a grade 3 diarrhea event (which in both patients, persisted despite dose reduction), the remaining grade 3 events were reversed after undertaking one level of dose reduction. However, no further escalation to 250 mg/bid was attempted. Thus, the RP2D was defined as 2.5 mg of letrozole combined with nintedanib (200 mg/bid).

All three patients in level 1 decided to continue treatment through the optional phase. Two of those patients completed 6 months of dosing without a significant toxic event, whereas the other patient withdrew her consent after 3 cycles because of prolonged grade 2 asthenia. Among the patients enrolled in level 2, 4 patients chose not to continue treatment after the first cycle (1 because of an investigator’s decision after diagnosing grade 3 high blood pressure; the other 3 patients did not report any particular reason). One additional patient was not offered continuation by the investigator. The remaining 11 patients decided to continue through the optional phase and completed a median number of 6 cycles (2 cycles: 1 patient; 3 cycles: 1 patient; 4 cycles: 3 patients; 6 cycles: 6 patients). The overall median treatment duration was 112 days (range, 15–175 days).

### Pharmacodynamics

The following two pharmacodynamic parameters were investigated in order to ascertain whether each agent was exerting its expected effect, despite any pharmacokinetic changes induced by concurrent administration: (1) plasma FGF23 increase on day 15 versus day 1 (secondary to FGFR1 inhibition) and (2) the change in 17-B-estradiol level on day 15 versus day 1. Whereas we expected to observe an increase in plasma FGF23 concentrations, we expected that 17-B-estradiol would remain below its LLD. Estradiol was also measured during the last dosing cycle and at the EOT visit.

**Fig. 2:**
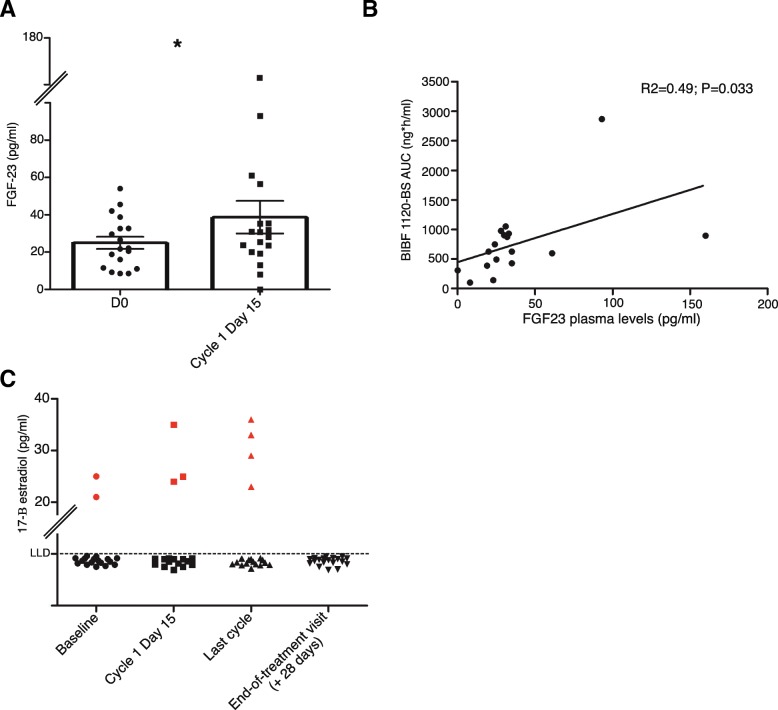
Pharmacodynamic parameters. **a** Change of FGF23 plasma concentration from baseline to day + 15 in level 2 patients. Horizontal error bars: standard error. The average concentration (columns) shifted from 24.9 pg/mL (baseline) to 38.7 pg/mL (d + 15). Each dot represents the value of a single patient. *P < 0.05 (Wilcoxon). **b** Dot plot showing the relationship between BIBF1120-BS AUC and FGF23 on day 15 at the individual patient level. **c** Change of 17-B-estradiol levels from baseline to day + 15, the first day of the last cycle, and at the EOT visit. Each gray dot represents one patient with 17-B-estradiol levels below the LLD. Red dots represent the individual 17-B-estradiol levels of those patients that showed a concentration above the LLD. Regarding the patients below the LLD, although the exact levels might be lower, all of them are plotted at the level of *Y* = 5 pg/mL which was our LLD

Figure 2a depicts the average FGF23 plasma concentrations of patients in the expansion cohort. There was an approximate 50% increase in FGG23 levels on day 15 versus day 1 (38.7 pg/mL on day 15 versus 24.9 pg/mL on day 1; *P* = 0.021). Figure 2b shows the relationship between BIBF1120-BS exposure and blood FGF23 levels. In general, on day 15, higher BIBF1120-BS AUC values were found to be correlated with higher FGF23 levels (*R*^2^ = 0.49; *P* = 0.033).

All but two patients (87.5%) had undetectable 17-B-estradiol levels on day 1 (Fig. 2c). Interestingly, three (18.7%) and four (25%) patients had 17-B-estradiol levels above the LLD on day 15, and also during the last cycle, which is equivalent to 81.2% and 75% of the patients having undetectable 17-B-estradiol levels, respectively. All patients (100%) had undetectable 17-B-estradiol levels 4 weeks after ceasing nintedanib administration (Fig. 2c).

### Toxicity

All patients experienced at least one grade 1 toxic event. No grade 4 toxicity or toxic deaths were registered. Table 2 lists the toxic events that were registered in at least 10% of the patients, and those events are categorized by being causally associated with nintedanib or letrozole. The majority of side effects were grade 1/2. The grade 3 increase in liver enzymes was transient and restricted to the first cycle in all cases, with the exception of one patient who required a nintedanib dose reduction, experienced persistent grade 3 toxicity in cycle 4, and decided to withdraw consent. The three grade 3 diarrhea events were successfully managed with nintedanib dose reduction and loperamide in two cases, whereas the third patient decided to interrupt treatment after the third cycle. That patient’s diarrhea was resolved at the end-or-treatment visit. Regarding hypertension, one patient (dose level 2, expansion cohort) was already taking two agents for adequate blood pressure control and was thus classified as grade 3 hypertension. That patient did not require dose-adjustment or any change of her anti-hypertensive medications. The other patient with grade 3 hypertension had not been previously diagnosed with hypertension but presented with a systolic blood pressure of 165 on day 28; the investigator considered that the patient had completed the study’s treatment phase and would not obtain additional benefits from continued participation and treatment. That patient was removed from the study and constituted the DLT observed among the first three patients recruited in level 2. No grade 3 toxic events were attributed to letrozole.

**Table 2:** Adverse events graded according to NCI CTC AE V.4.03 registered in at least 10% of the patients related to the study drugs

	Level 1	Level 2			Total
	Grades 1–2	Grade 3	Grade 1–2	Grade 3	
Nintedanib-related event					
Diarrhea	1/3 (33%)	0/3 (0%)	9/16 (56%)	3/16 (18.8%)	13/19 (68%)
Nausea	2/3 (66.6%)	0/3 (0%)	9/16 (56%)	0/16 (0%)	11/19 (58%)
Vomiting	2/3 (66.6%)	0/3 (0%)	9/16 (56%)	0/16 (0%)	11/19 (58%)
Asthenia	1/3 (33%)	1/3 (33%)	9/16 (56%)	0/16 (0%)	11/19 (58%)
Elevated GGT	0/3 (0%)	0/3 (0%)	4/16 (25%)	4/16 (25%)	8/19 (42%)
Elevated AST	1/3 (33%)	0/3 (0%)	4/16 (25%)	2/16 (12.5%)	7/19 (36.8%)
Elevated ALT	0/3 (0%)	0/3 (0%)	4/16 (25%)	2/16 (12.5%)	6/19 (31.5%)
Hyporexia	0/3 (0%)	0/3 (0%)	3/16 (18.8%)	0/16 (0%)	3/19 (15.8%)
Dysgeusia	1/3 (33%)	0/3 (0%)	2/16 (12.5%)	0/16 (0%)	3/19 (15.8%)
Hypertension	0/3 (0%)	0/3 (0%)	0/16 (0%)	2/16 (12.5%)	2/19 (10.5%)
Headache	1/3 (33%)	0/3 (0%)	1/16 (6.2%)	0/16 (0%)	2/19 (10.5%)
Letrozole-related event					
Asthenia	2/3 (66.6%)	0/3 (0%)	8/16 (50%)	0/16 (0%)	10/19 (52.6%)
Arthralgia	1/3 (33%)	0/3 (0%)	7/16 (43.8%)	0/16 (0%)	8/19 (42%)
Hot flushes	1/3 (33%)	0/3 (0%)	6/16 (37.5%)	0/16 (0%)	7/19 (36.8%)

No patient required a nintedanib dose reduction in level 1. In level 2, four patients required one dose reduction (two because of grade 3 diarrhea [resolved], one because of prolonged grade 2 asthenia and grade 3 diarrhea [non-resolved], and one because of grade 3 ALT elevation [resolved], and one patient required two dose reductions [the first time because of grade 3 AST/ALT elevation and second time because of grade 3 GGT elevation [non-resolved]]). No patient required a letrozole dose reduction. Overall, toxicity was well managed with supportive measures and reversed after discontinuation of treatment. No severe adverse events were registered. Other toxicities commonly attributed to multi-tyrosine-kinase inhibitors such as skin rash or palmar-plantar erythrodysesthesia were observed in 6.5% (grade 1) and 0% of the patients, respectively.

### Pharmacokinetics

**Fig. 3:**
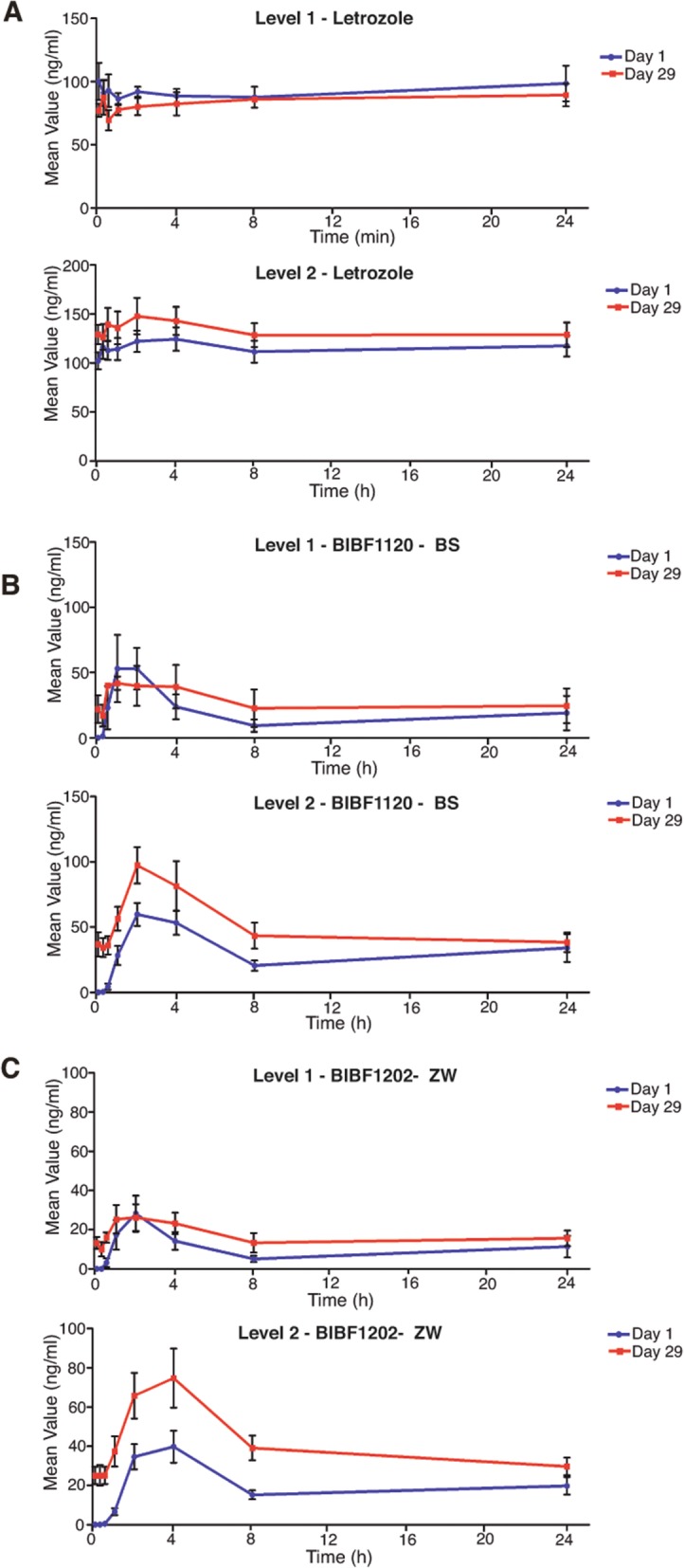
Pharmacokinetic parameters. **a** Plasma letrozole levels at both dose levels, comparing in each the mean plasma levels in nanograms per milliliter on day 29 versus 1 along the 24-h course. **b**, **c** The same as in **a** for both active nintedanib metabolites BIBF1120-BS and BIBF1202-ZW, respectively. *N* = 3 in level 1; *N* = 16 in level 2. Error bars: standard error

On day 1, all patients had taken letrozole for at least 28 days and had steady-state plasma concentrations. The introduction of nintedanib induced an increase in the mean Cmax letrozole plasma concentrations (Fig. 3a) and mean letrozole AUC values (Table 3) in dose level 2. In addition, continuous nintedanib administration induced a further increase in both parameters, according to the values recorded for letrozole pharmacokinetic parameters on day 29 (Fig. 3a, Table 3).

**Table 3:** Day 1 and day 29 pharmacokinetic parameters (letrozole and nintedanib)

LET	Mean *C*_max_ (ng/ml)	Mean *T*_max_ (h)	Mean AUC_0-last_ (ng-h/ml)	NINT	Mean *C*_max_ (ng/ml)	Mean *T*_max_ (h)	Mean AUC_0-last_ (ng-h/ml)
Level 1 day 1	100.2	0	2204	BS level 1 day 1	51	1.9	339
Level 1 day 29	89.4	4	2059	BS level 1 Day 29	50	1.1	519
Level 2 day 1	124.5	4	2782	BS level 2 day 1	65	2.0	599
Level 2 day 29	148.0	2	3170	BS level 2 day 29	92	2.0	941
				ZW level 1 day 1	24	1.9	200
				ZW level 1 day 29	28	1.1	367
				ZW level 2 day 1	41	3.3	413
				ZW level 2 day 29	67	3.0	824

*LET* letrozole, *NINT* nintedanib, *BS* BIBF1120-BS, *ZW* BIBF1202-ZW

Conversely, there was no period during the trial when nintedanib was administered as monotherapy. As expected, the values for the nintedanib pharmacokinetic parameters were higher on day 29 than on day 1 and, as expected, were higher in the level 2 patients compared to the level 1 patients (Table 3, Figs. 3b and c). The mean plasma BIBF1120-BS and BIBF1202-ZW concentrations were almost twofold higher during their steady states (day 29) when compared with their plasma concentrations on day 1 (level 2; Fig. 3b, c). Whereas this increase in nintedanib concentration seen on day 29 versus day 1 has been previously described [19], the concurrent administration of letrozole led to almost threefold increases in AUC and mean plasma concentration.

## Discussion

Phase 0 trials have the ability to gather data that can be used to optimize time and resources during the drug development process by allowing go/no-go decisions to be made in a relatively short time period and at a controlled cost [30]. In this phase 0/1 trial, in addition to studying the toxicity and long-term tolerability of the combination of letrozole plus nintedanib, we sought to determine whether both drugs were exerting their expected pharmacodynamic effect, even in the presence of significant pharmacokinetic interactions.

In the rapidly evolving field of biomarker-driven disease segmentation, FGFR1 has been investigated as a potential driver of several hormone-refractory/resistant clinical conditions that can be included within the overall category of hormone receptor-positive breast cancer [2–4]. Preclinical data suggest that the optimal treatment for this disease cluster would consist of a hormone-blocking agent combined with an FGFR inhibitor [2, 7]. Eventually, registration trials aimed at improving the disease control rates in a metastatic setting or decreasing the relapse rate of this disease cluster in an adjuvant setting will require time-consuming and expensive clinical trials that involve prolonged concurrent administration of an FGFR inhibitor plus a standard hormonal blockade. Although various molecules with selective and non-selective activity in regulating the FGFR family have been developed, data concerning their use in combination with hormonal agents are scarce [6]. A clinical trial that combined fulvestrant with the multikinase inhibitor lucitanib was prematurely terminated; however, the patients in that trial had been previously exposed to fulvestrant, and toxicity limited the administration of lucitanib at full doses [31]. Another recent clinical trial that combined fulvestrant with the multikinase inhibitor dovitinib or a placebo showed promising signs of clinical activity in a FGFR-amplified breast cancer population [32]. However, to the best of our knowledge, this is the first trial to demonstrate effective inhibition of both pathways of interest (17-B-estradiol synthesis and FGFR1 signaling).

Clinical trials with other FGFR inhibitors have reported variable degrees increased FGF23 concentrations. These increases ranged from ~ 20 to ~ 50% at the RP2Ds for JNJ-42756493 [8] and BGJ398 [9], respectively. To our knowledge, there is no previous evidence for FGFR1 being modulated by nintedanib, when administered at standard doses. Here, we have provided proof for this modulation by observing an average ~ 50% increase FGF23 levels on day 15 versus 0 (Fig. 2a). Despite this increase, a number of patients did not experience that result. However, a dose-response effect was observed between nintedanib exposure and FGF23 levels (Fig. 2b). The reason why some patients achieve greater drug exposure than others with the same drug dosages needs to be explored in large pharmacogenomic studies but is commonly observed in phase I trials. Regardless, the dose-response effect (Fig. 2b) supports the FGFR1 inhibitory properties of nintedanib. Regarding 17-B-estradiol, despite observing an increase in letrozole AUC values and mean plasma concentrations in dose level 2 versus level 1 (Table 3, Fig. 3a), we also observed a greater but non-statistically significant number of patients with detectable levels of 17-B-estradiol (Fig. 2c). Early letrozole trials reported a > 95% suppression of aromatase activity and a general decrease in 17-B-estradiol levels [33, 34]. However, the true percentage of patients who achieve suppression below the LLD in large populations is unknown, because the large letrozole phase III trials conducted to date have not included that endpoint [35–38]. Two studies addressed the degree of 17-B-estradiol suppression in response to anastrozole (*n* = 191) [39], letrozole (*n* = 241), or exemestane (*n* = 228) [40] after various treatment periods (4 weeks to 6 months). In the anastrozole study, it was found that 83% of the patients displayed plasma 17-B-estradiol concentrations below the LLD [39]. In the second study, 89% and 86.9% of the patients had suppressed levels of 17-B-estradiol after treatment with letrozole and exemestane, respectively [40]. An additional study randomized patients to 3 months of letrozole followed by 3 months of anastrozole and then compared them to patients randomized to the opposite sequence. That study found a greater suppression of 17-B-estradiol levels among patients who received letrozole, with only 2% of patients being above the LLD (compared to 37% of the patients who received anastrozole) [41]. The number of patients with 17-B-estradiol levels above the LLD was not significantly higher in our study; however, the lack of testing at different time points in the other studies [39, 40] limits our ability to conclude whether the fact that 17-B-estradiol suppression was observed in 100% of the patients after nintedanib cessation is significant. An additional limitation to our study is that 17-B-estradiol was detected with a clinical assay that was not sufficiently sensitive to detect residual levels, while in letrozole treatment compared to the tenfold more sensitive assay used by Dixon and colleagues [41]. Regardless, the fact that across all the different studies, up to 15% of the patients did not show complete 17-B-estradiol suppression, and taking into account that large numbers of patients receive aromatase inhibitors in an adjuvant setting, the insufficient suppression of 17-B-estradiol levels might be a significant factor contributing to the non-negligible number of metastatic relapses. Careful monitoring of 17-B-estradiol levels should be combined with individual pharmacogenomics data in a future prospective study designed to improve the individualized treatment of hormone receptor-positive advanced breast cancer.

Although multikinase inhibitors are associated with significant toxicity [42–44], nintedanib has consistently shown long-term acceptable tolerance when administered at 200 mg/bid [13, 14, 45, 46]. Our study included a 28-day period for DLT assessment and that short-term assessment period was used to determine the RP2D. Furthermore, the short-term toxicities seen with nintedanib were mild (Table 2), and allowed escalation to the currently recommended standard dose of nintedanib, despite concurrent letrozole administration. However, besides the fact that most of the toxic events were grade 1/2 (Table 2), toxicities such as diarrhea, nausea, vomiting, or fatigue clearly impacted the long-term tolerability of the treatment regimen, and due to various reasons associated with sustained, low-grade toxicity, only 6 out of 16 patients in dose level 2 completed all 6 treatment cycles. Also, the previous administration chemotherapy to 1/3 of the patients enrolled in the study may have influenced the long-term tolerance to the dosing regimen. The importance of collecting and evaluating long-term tolerability data, and considering it when designing long treatment periods such as those in an adjuvant stage, must be underscored. Conversely, no patient decided to withdraw their consent and abandon our trial because of toxicities allegedly related to letrozole, although it should be highlighted that this trial was conducted in patients who had been shown to tolerate letrozole. Interestingly, we did not notice meaningful rates of toxicities commonly associated with specific FGR1 inhibitors, such as hyperphosphatemia, mucositis, and osteoarticular toxicity [6].

Pharmacokinetic interactions may explain the long-term toxicity observed in our study. Although nintedanib pharmacokinetic parameters were assessed only during cycle 1, the comparison made between day 29 and day 1 clearly suggested drug accumulation. Both letrozole and nintedanib are metabolized by Cyp3A4, and in line with our results, it appears that this interaction is clinically significant (Table 3; Fig. 3b, c). The BIBF1120-BS AUC values on day 29 were almost twofold higher than those recorded on day 1 and were two to threefold higher than the steady-state values reported in phase I studies of nintedanib monotherapy [28], nictedanib combined with pemetrexed [27], or paclitaxel plus carboplatin [47]. Regarding letrozole, although we observed the same effects (an increase on day 29 versus day 1 and in level 2 versus 1), the plasma letrozole levels were similar to those observed in healthy subjects treated with letrozole [48]. Those levels were also in line (or lower) with letrozole levels previously reported in cancer patients enrolled in monotherapy [33] and combination studies [34, 49, 50]. This may explain the lack of dropouts due to letrozole-related toxicity. Because exemestane [51] and anastrazole [52] are also metabolized by Cyp3A4, it might be difficult to avoid nintedanib accumulation. Although other hormonal inhibitors exist, pharmacodynamic determinations of activity for tamoxifen or fulvestrant would imply serial sampling of tumor tissue and complex quantitation procedures given their mechanisms of action, complicating the conduction of a phase 0 trial.

The following facts should be taken into consideration when planning to conduct a clinical trial in an adjuvant or metastatic setting where the patients would receive this type of combination therapy for a prolonged period of time: (1) we did not observe significant differences between the FGF23 and/or letrozole levels in dose levels 1 and 2, although the small number of patients in level 1 precludes concluding that FGFR1 and aromatase were effectively modulated at a dose of 150 mg/bid; (2) our pharmacokinetic data suggest that not all patients achieved effective FGFR1 inhibition; however, the likelihood of achieving effective inhibition is directly related to the nintedanib AUC value; (3) a pharmacokinetic interaction occurs between letrozole and nintedanib that results in nintedanib accumulation in the long term; and (4) in the long term, the combination of nintedanib plus letrozole produces a moderate rate of non-tolerable toxicity. Based on these findings, we propose proceeding according to the following algorithm: a patient should start at 200 mg/bid of nintedanib plus 2.5 mg of letrozole. The FGF23 level should be determined after 2 weeks and compared with the level at baseline. If a > 25% increase in FGF23 is observed, and the treatment regimen is tolerated, the patient should continue at the same level. If a < 25% increase in FGF23 is observed, the patient should stop taking the drug because of its uncertain pharmacodynamic effect. In the first case, if non-tolerable toxicity is observed, one dose reduction (to 200 mg/bid of nintedanib plus 2.5 mg of letrozole) should be attempted, and FGF23 patient’s level should be re-determined 2 weeks later. The final decision on whether to continue the treatment should depend on whether an effective pharmacodynamic modulation can still be observed.

In conclusion, our study met its primary endpoint by showing pharmacodynamic evidence of FGFR1 modulation at the RP2D and no significant impact on the ability of letrozole to suppress 17-B-estradiol activity. The RP2D was defined as 200 mg/bid of nintedanib combined with 2.5 mg/day of letrozole. The tolerance to this dosing regimen was acceptable in the short term, although in the long term, the cumulative and durable incidence of low-grade toxicities such as diarrhea and asthenia led to patient attrition and a meaningful patient dropout rate. These toxic effects may have been caused by a pharmacokinetic interaction between letrozole and nintedanib that resulted in increased plasma concentrations of the latter drug. However, this problem can be solved by nintedanib dose reductions “on demand,” and other methods for solving this problem can be guided by making serial FGF23 determinations, given the direct correlation between nintedanib exposure and FGF23 levels. Data concerning the clinical efficacy of a FGFR1 inhibitor used in combination with hormonal treatment in cases of breast cancer are still scarce. Taken together, a clinical trial testing the efficacy of nintedanib plus letrozole, and restricted to metastatic FGFR1-amplified hormone receptor-positive breast cancer patients, would be the reasonable next step in such clinical investigations.

## Conclusion

The combination of nintedanib (200 mg/bid) plus letrozole (2.5 mg/d) effectively inhibited both FGFR1 and aromatase in breast cancer patients, as evidenced by plasma FGF23 and 17-B-estradiol levels. This combination produced a moderate rate of non-tolerable toxicity in the long term; thus, in order to conduct a clinical trial that includes prolonged treatment in an adjuvant or metastatic setting, a careful toxicity assessment combined with FGF23 monitoring should be incorporated into the study protocol. Given the increased use of CDK inhibitors in standard of care protocols and the limited activity of selective FGFR inhibitors in FGFR1-amplified breast cancer in basket trials, based on the safety and pharmacodynamic data found on this study, the combination could be further evaluated in molecularly selected patients the moment there is a compelling clinical/preclinical rationale supporting a specific target niche.

## Abbreviations

ALT: Alanine aminotransferase
AST: Aspartate aminotransferase
AUC: Area under the curve
CT: Computed tomography
DLT: Dose-limiting toxicity
ECOG: Eastern Cooperative Oncology Group
EOT: End-of-treatment visit
FGF: Fibroblast growth factor
FGF23: Fibroblast growth factor 23
FGFR1: Fibroblast growth factor receptor 1
LLD: Lower limit of detection
LVEF: Left ventricular ejection fraction
MRI: Magnetic resonance imaging
NCI CTC AE: National Cancer Institute Common Toxicity Criteria for Adverse Events
PDGFR: Platelet-derived growth factor receptor
RP2D: Recommended phase-II dose
SNVs: Single-nucleotide variants
TKI: Tyrosine-kinase inhibitors
ULN: Upper normal limit
VEGFR: Vasculo-endothelial growth factor
